# Proximal interruption of the pulmonary artery: A review of radiological findings

**DOI:** 10.3389/fped.2022.968652

**Published:** 2022-10-31

**Authors:** Ming-Jie Zhang, Ya-Xian Cao, Ning Zhou, Rui Wang, Hui-Ying Wu, Xiao-Chun Zhang

**Affiliations:** Department of Radiology, Guangzhou Women and Children’s Medical Center, Guangzhou, China

**Keywords:** computed tomography, congenital cardiovascular abnormality, pulmonary artery, interruption, absence

## Abstract

**Objectives:**

Proximal interruption of the pulmonary artery (PIPA) has various clinical manifestations. This review focused on and summarized the clinical and radiological features of PIPA, based on relevant literature studies.

**Methods:**

The study included a total of 25 PIPA cases in the Guangzhou Women and Children's Medical Center between January 2015 and December 2021. Conventional chest photographs and chest computed tomography angiography (CCTA) of patients with PIPA were analyzed and summarized.

**Results:**

The radiological results showed that 17 cases were right-sided and 8 cases were left-sided PIPA. Additionally, the percentage of pulmonary hypoplasia on the affected side was 44%, 36% for pulmonary hypertension, 28% for the mosaic sign, 20% for subpleural cystic lucency shadow, 20% for subpleural serrated shadow, 20% for collateral vessel thickening, 16% for subpleural band-like parenchyma, 12% for pneumonia, and 56% for patent ductus arteriosus.

**Conclusion:**

The clinical manifestations of PIPA are non-specific. Awareness of this anomaly, based on radiological manifestations, particularly those observed on CCTA images, is important for ruling out alternative diagnoses and implementing appropriate management.

## Introduction

According to Richardson (1), the anatomic classification of aortopulmonary defects can be divided into three types as follows. (1) Type I defects, which occur between the ascending aorta and the main pulmonary artery just above the sinus of Valsalva. (2) Type II defects are more cephalad-oriented, between the ascending aorta and the origin of the right pulmonary artery from the main pulmonary artery. (3) Type III defects have an anomalous origin from the right pulmonary artery from the aorta. Proximal interruption of the pulmonary artery (PIPA), a rare congenital pulmonary artery abnormality, belongs to the Type I category (2) and can occur on its own or with other congenital cardiovascular abnormalities. Both pulmonary arteries may be involved, but the condition generally occurs more commonly on the right side. The estimated incidence of PIPA, which was first reported by Doring in 1914, is approximately 1/200,000 (3–5). Other authors reported the incidence of PIPA without cardiac abnormalities as being 1/300,000–1/200,000 (6, 7). As reported by Bockeria (8), 237 of 352 patients with PIPA had cardiovascular abnormalities.

There are no significant symptoms or signs in most PIPA cases. Potential clinical symptoms include exercise intolerance, shortness of breath on exertion (SBE), recurrent pulmonary infections, hemoptysis, and pulmonary hypertension. When complicated by cardiac abnormalities, PIPA may manifest as symptoms and signs associated with cardiovascular diseases, such as cyanosis and heart failure. It is more difficult and challenging to make a clinical diagnosis of PIPA in infants and children; however, radiological examination, including routine chest photography and chest computed tomography angiography (CCTA), can provide significant valuable information for a definitive diagnosis of PIPA. The present study included a group of 25 infants and children with PIPA, and the condition's clinical and radiological features were summarized.

## Materials and method

A total of 25 patients (12 female and 13 male, average age, 10.2 months) with PIPA, confirmed by computed tomography (CT), echocardiography, or surgery in the Guangzhou Women and Children's Medical Center between January 2015 and December 2021 were included in this study.

Echocardiography and CCTA were performed for all the patients. A Philips Brilliance CT 64 scanner was used to do so. Sedation was required for children aged between 1 month and 4 years. The need for sedation in children above 4 years old depended on their level of cooperation. The lower limb vein was the preferred site for the venous indwelling needle to avoid artifacts caused by high-density contrast agents in the superior vena cava (9, 10). The size of the venous indwelling needle mainly depended on the patient's weight; 24G or 22G venous indwelling needles were most commonly used. The total dosage of the contrast agent was 1.5–2 ml/kg; if this was insufficient, it was diluted with normal saline to maintain a bolus duration of 15–25 s, but the total amount of liquid did not exceed 10 ml/kg (11, 12). The scan could be started 1–3 s after the contrast agent was fully injected. The tube voltage was 80 kV, and the tube current was automatically adjusted following the “as low as reasonably achievable” principle.

The clinical symptoms and radiological examination data of all the patients were reviewed to summarize the clinical and radiological features of PIPA.

## Results

### Clinical manifestations

There were 15 patients without clinical symptoms; 6 patients had a cough and shortness of breath, 2 patients had hemoptysis, and 2 patients had cyanosis. There were 8 cases of cardiovascular abnormalities, including 2 cases of pulmonary vein stenosis (both of which were right-sided PIPA with left pulmonary vein stenosis, means on the opposite side), one case of complete atrioventricular canal (CAVC) defect/double outlet right ventricle (DORV)/single ventricle (SV)/pulmonary stenosis (PS), one tetralogy of Fallot (TOF) case, one case with an aortopulmonary septal defect (APSD), one case with an atrial SD (ASD), one vascular ring case (left aortic arch with a circumflex aorta and aberrant right subclavian artery), and one case showing a coronary artery of anomalous origin.

### Radiological findings

There were 17 cases of right-sided PIPA (16 with a left-sided aortic arch and 1 case with a right-sided aortic arch) and 8 cases of left-sided PIPA (6 with a right-sided aortic arch and 2 with a left-sided aortic arch). There were also 12 cases of unilateral patent ductus arteriosus and 2 cases of bilateral patent ductus arteriosus, as well as 17 cases of isolated PIPA and 8 cases of PIPA with cardiovascular abnormalities (6 with right-sided PIPA and 2 with left-sided PIPA). There were 11 cases of pulmonary hypoplasia, 9 cases of pulmonary hypertension, 7 mosaic sign cases, 5 collateral vessel cases, 5 cases of subpleural lucency shadow, 5 subpleural serrated shadow cases, 4 cases with a subpleural parenchymal band, and 3 cases of pneumonia.

### Echocardiography and surgical results

Echocardiography indicated the absence of the pulmonary artery in all cases, which was confirmed in 11 of 25 patients by surgery. The remaining 14 cases did not undergo surgery.

## Discussion

### Clinical symptoms

Proximal interruption of the pulmonary artery can be divided into two categories, based on its clinical manifestations as follows: (1) isolated PIPA, without other cardiovascular abnormalities (excluding patent ductus arteriosus); (2) complex PIPA, with a variety of different cardiovascular abnormalities.

Many researchers believe isolated PIPA to often be asymptomatic in early childhood, followed by symptoms that include exercise intolerance, SBE, recurrent pulmonary infections, pulmonary hypertension, or hemoptysis (13, 14–16), as the patient ages. However, as reported by Harkel et al. (17), isolated PIPA is asymptomatic in only 13% of patients. In complex PIPA accompanied by other cardiovascular abnormalities, clinical symptoms are associated with the pathophysiological manifestations of the abnormalities.

### Dyspnea on exertion

Dyspnea on exertion may be associated with ipsilateral pulmonary hypoplasia, insufficient blood flow in the pulmonary artery, recurrent pulmonary infections, pulmonary parenchymal cystic changes and fibrosis, pulmonary arterial hypertension, and other pulmonary function impairments.

### Recurrent pulmonary infections

The pathogenesis of recurrent pulmonary infections remains unclear. It may be caused by multiple factors, including reduced pulmonary blood perfusion on the affected side, the mismatching of alveolar ventilation/perfusion, and a decreased airway mucosal defense (17). In addition, bronchoconstriction, a reduced release of inflammatory cells caused by alveolar hypocapnia, and mucociliary dysfunction of the trachea and bronchus can all contribute to recurrent pulmonary infections (5, 18, 19).

### Hemoptysis

Hemoptysis may be caused by excessive systemic–pulmonary collateral circulation flow (17, 18, 20, 21), and approximately 10% of patients with PIPA suffer from hemoptysis due to the rupture of thin-walled and excessively enlarged collateral vessels (22). Hemoptysis is typically mild and can remain self-limited for a long time (23); however, massive hemoptysis may occur in rare cases, for which emergency pulmonary resection or systemic–pulmonary collateral vessel embolization will be required (22).

Bronchiectasis in the affected lung is also a cause of hemoptysis. Although recurrent pneumonia is an important cause of bronchiectasis, studies have suggested that pulmonary artery interruption also affected the development and growth of the affected lung and bronchi, which may lead to bronchiectasis (20, 24). Pulmonary fibrosis may also be a cause of bronchiectasis, and Sage et al. (25) reported that patients with PIPA also had subpleural pulmonary fibrosis.

### Concomitant cardiovascular abnormalities

Cases of PIPA are primarily isolated and seldom complicated by congenital heart disease, which only accounts for <1% of cases (13, 15). The incidence of PIPA cases without cardiac abnormalities ranges between 1/300,000 and 1/200,000 (6, 7). According to statistics conducted by Bockeria (8), the incidence of PIPA with cardiovascular abnormalities was 237/352. The proportion of cardiovascular abnormalities in the group in the present study was 32% (8/25).

Right-sided PIPA occurs in isolation in most patients, without other cardiovascular abnormalities (26, 27). However, approximately 80% of patients with left-sided PIPA also have other congenital cardiovascular abnormalities, including TOF, ASD, and ventricular SD (17, 26, 28–30). Co-occurring cardiovascular abnormalities are an important influencing factor of prognosis. In the present study, 35% (6/17) of patients with right-sided PIPA and 25% (2/8) of patients with left-sided PIPA had cardiovascular abnormalities.

### Pulmonary arterial hypertension

Currently, pulmonary hypertension is defined as a mean pulmonary arterial pressure of 20 mm Hg or greater at rest (as confirmed by right-sided heart catheterization) (31).

Proximal interruption of the pulmonary artery may cause pulmonary hypertension and right-sided heart failure (17). The pathogenesis of pulmonary hypertension may be increased blood flow in the collateral arteries of the affected lung, remodeling of the pulmonary arterioles, and increased vascular resistance, which will gradually give rise to pulmonary hypertension (32). Furthermore, the significant increase of pulmonary blood flow to the unaffected side will lead to an increase in pulmonary vascular resistance, which is also an important cause of pulmonary hypertension.

Based on the literature review, pulmonary hypertension occurred in 19%–44% of patients with PIPA (6, 16, 17, 20). The proportion of pulmonary hypertension in the group in the present study was 36% (9/25). Pulmonary hypertension is currently considered a critical sign and the most important factor for determining patient prognosis (6, 24).

### Conventional chest radiography and computed tomography angiography findings

#### Pulmonary hypoplasia

**Figure 1:**
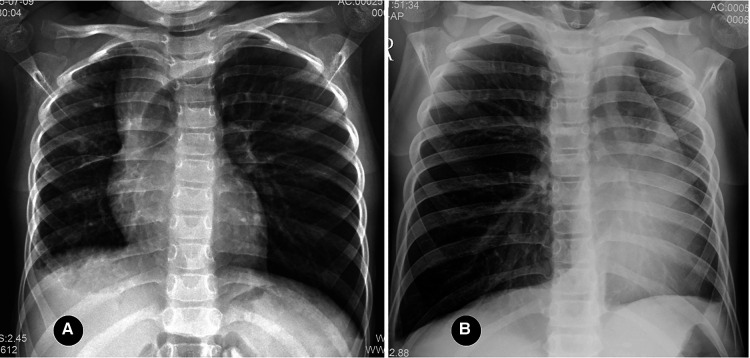
(**A**) right proximal interruption of the pulmonary artery (PIPA) and right-lung hypoplasia in a 4-year-old female patient. An anteroposterior radiograph of the chest shows a small volume of the right thoracic cage, the small volume of the right lung, the small hilum of the right lung, and slightly increased lucency of the right lung field. The left lung was hyperinflated, the mediastinum shifted to the right, and the diaphragm was slightly elevated on the right. (**B**) left-sided PIPA and left-lung hypoplasia in a 7-year-old male patient. An anteroposterior radiograph of the chest shows a reduced volume of the left thorax and lung, a mildly dense left lung field, and an increased volume of the right lung. The mediastinum is shifted to the left.

The relatively common signs of pulmonary hypoplasia include varying levels of decrease in the volume of the affected thorax and lung, small hilar opacity, increased lung field lucency, excessive expansion of the unaffected lung, mediastinum displacement to the affected side, and varying levels of elevation of the diaphragm on the affected side (see Figure 1A). Pulmonary hypoplasia accounted for 44% of cases in the group in the present study. Furthermore, the reticulated shadow in the lung field of the affected side represented the transpleural systemic–pulmonary collateral vessels.

Although the lucency of the PIPA-affected lung field typically increases, researchers have reported that the lucency can also be equal to or slightly higher than that of the unaffected side (see Figure 1B). This was believed to be because the PIPA-affected lung included fewer and smaller alveoli and because low oxygen partial pressure may be an important stimulating factor for the development of alveoli in the initial postnatal eight years (33).

#### Proximal interruption of the pulmonary artery

In general, congenital pulmonary artery abnormalities can be classified into the following types: primary pulmonary artery stenosis or dilation, co-occurring abnormalities, and pulmonary artery origin/route or connection abnormalities (13, 34). Proximal interruption of the pulmonary artery falls into the category of primary pulmonary artery stenosis, and the intrapulmonary arteries on the PIPA-affected side are typically intact and open (26, 35, 36).

**Figure 2:**
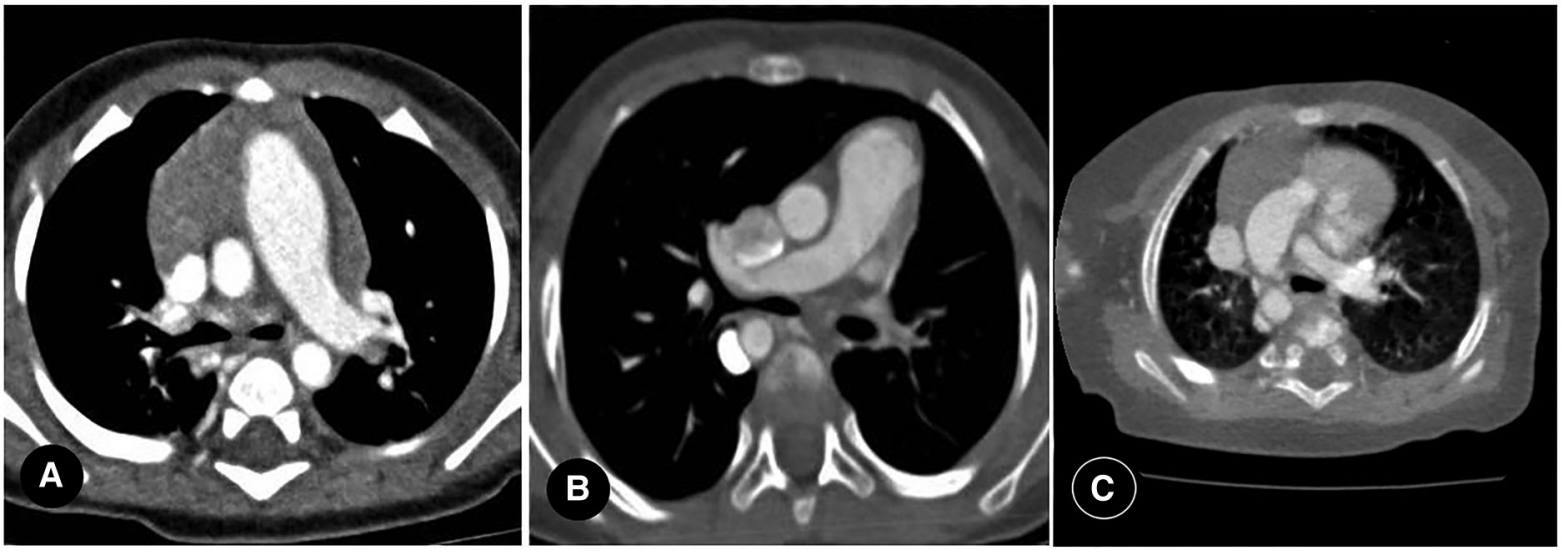
(**A**) right proximal interruption of the pulmonary artery (PIPA) in a 4-month-old male patient. A transverse view of the chest computed tomography angiography (CCTA) shows no indication of the mediastinal segment of the right pulmonary artery, no pulmonary artery confluence, and a right-sided hilar artery. The right internal pulmonary artery branches were open. Left-sided aortic arch. (**B**) left-sided PIPA in a 19-month-old female patient. A transverse view of the CCTA shows no indication of the mediastinal segment of the left pulmonary artery, no pulmonary artery confluence, and a left-sided hilar artery. The left internal pulmonary artery branches are open. Right-sided aortic arch. (**C**) right PIPA with a right-sided aortic arch in a 6-month-old male patient. A transverse view of the CCTA shows the absence of the right pulmonary artery with a right-sided aortic arch.

Primarily, PIPA manifests as the complete absence of the mediastinal segment of the unilateral pulmonary artery. That is, the pulmonary artery segment between the expected pulmonary artery bifurcation and the affected pulmonary hilum is not shown at all, and the main pulmonary artery continues into the contralateral pulmonary artery on its own. This can occur on either the left or right side (see Figures 2A–C) but is generally believed to be more common on the right. Approximately 68% of PIPA cases in the present study were on the right side, and 32% were on the left. However, another group of studies showed that left-sided PIPA occurred in 6/8 patients (24).

In addition, the proximal segment of the pulmonary artery in the affected side of some patients with PIPA appeared as a short blind tube, but most of them were interrupted within 1 cm of the origin site, and the lumen was relatively smaller (28). The pulmonary trunk and the unaffected pulmonary artery are typically enlarged to varying degrees, indicating pulmonary hypertension, but the distribution of the intrapulmonary branches in the affected pulmonary artery remains normal. Right-sided PIPA generally occurs in isolation, while left-sided PIPA is frequently complicated by other cardiovascular abnormalities.

#### Patent ductus arteriosus and systemic–pulmonary collateral arteries

The ductus arteriosus of patients with PIPA is kept either open or closed, and the blood supply to the affected lung occurs either through the ductus arteriosus or the transpleural systemic–pulmonary collateral arteries (35, 37).

In the neonatal period, the affected lung typically receives arterial blood supply from the open ductus arteriosus. If this is closed, however, and the pulmonary artery is absent after birth, the ipsilateral intrapulmonary artery branch will have a reduced blood supply, leading to aggravation of the disease. Early surgical correction is recommended for ductus arteriosus-dependent circulation in infants because the ductus arteriosus often degenerates at the age of one month, resulting in hypoplasia of the intrapulmonary arterial branches, thereby reducing the possibility of surgical success (13).

**Figure 3:**
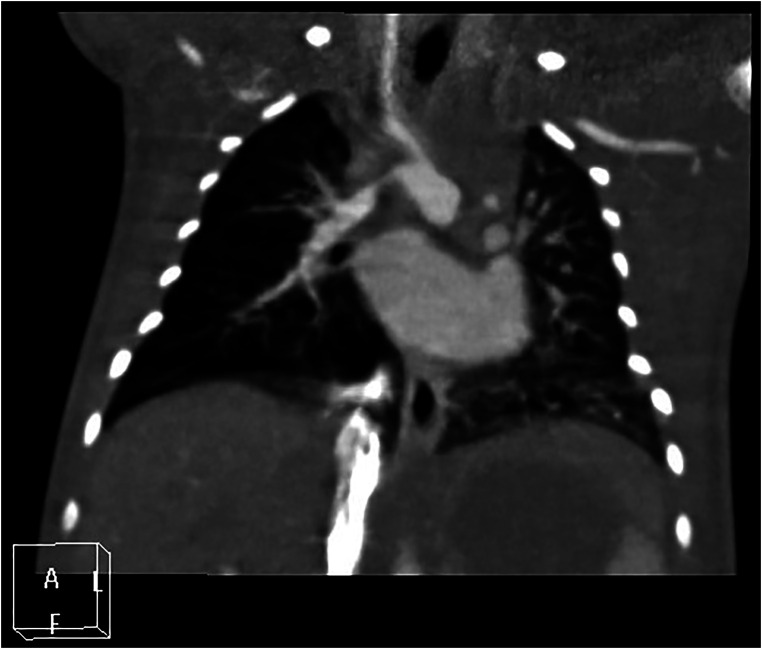
Right proximal interruption of the pulmonary artery, with left-sided aortic arch and right-sided patent ductus arteriosus in a 4-day-old female patient. A coronal view of the chest computed tomography angiography shows that the proximal right pulmonary artery is absent, and an open ductus arteriosus connects the proximal brachiocephalic trunk to the distal right pulmonary artery.

**Figure 4:**
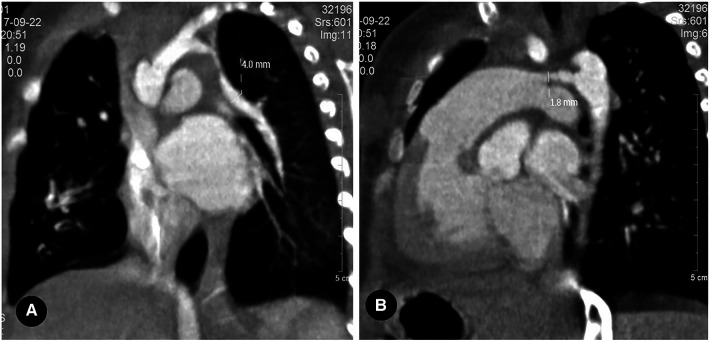
Left proximal interruption of the pulmonary artery with bilateral patent ductus arteriosus in a 4-month-old male patient. (**A**) a coronal view of the chest computed tomography angiography (CCTA) shows an open left ductus arteriosus connecting to the distal end of the left pulmonary artery. (**B**) an oblique coronal view of the CCTA shows an open right ductus arteriosus connecting the descending aorta to the proximal end of the right pulmonary artery.

There are differing opinions on the nature of the blood-supplying vessels connecting the proximal brachiocephalic trunk to the distal right pulmonary artery in patients with right-sided PIPA, where the debate centers on whether it is a right-sided arterial duct or a systemic–pulmonary collateral artery. In the context of embryonic development, the current authors suggest a greater likelihood that this is right-sided patent ductus arteriosus (see Figure 3). A bilateral open ductus arteriosus is also present in some special cases (see Figures 4A,B).

In older children with a closed ductus arteriosus, the pulmonary circulation is supplied by the bronchial artery and systemic collateral arteries, including the inferior phrenic, internal mammary, intercostal, subclavian, and even coronary arteries (13, 26, 35, 37).

**Figure 5:**
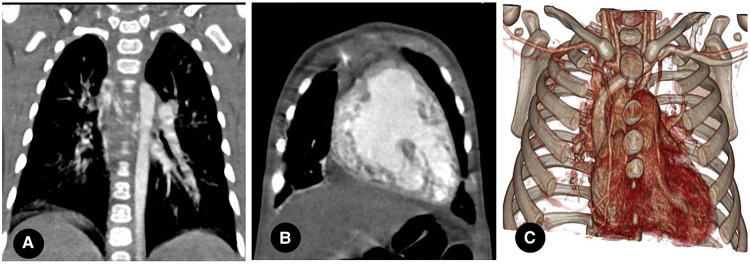
(**A**) right proximal interruption of the pulmonary artery (PIPA) in a 4-month-old male patient. A coronal view of the chest computed tomography angiography (CCTA) shows a dilated bronchial artery with small branches of the right internal pulmonary artery, a collateral circulation formed by the bronchial artery, and a right internal pulmonary artery segment. (**B**) right-sided PIPA in a 4-month-old male patient. A coronal view of the CCTA shows the dilation of the right inferior phrenic artery supplying blood to the right lower lung lobe through collateral arteries. (**C**) right-sided PIPA in a 4-month-old male patient. A virtual reality image shows the dilation and thickening of the right internal thoracic artery and the right subclavian artery and its branches.

Normal pulmonary circulation is a low-resistance, high-capacity system with approximately 1/10 systemic circulation blood flow resistance (31). Bronchial circulation accounts for approximately 1% of total cardiac output and delivers blood at approximately six times the pulmonary circulation pressure (38). Under normal circumstances, bronchial circulation only supplies nutrients and does not exchange gas. However, in patients with PIPA, bronchial circulation also participates in blood oxygen exchange because of the significant reduction in pulmonary blood flow on the affected side. The bronchial circulation vessels respond to pulmonary ischemia through hypertrophy and diameter enlargement, and the vascular networks of pulmonary and bronchial circulation exchange through connections between several microvessels. Moreover, a direct joining of other transpleural systemic collateral arteries (e.g., intercostal and intrathoracic arteries) to the pulmonary artery can also be achieved (see Figures 5A–C) (38–40). Some researchers posited that selected aortopulmonary collateral arteries have significant histological similarities with ductus arterioles (41).

**Figure 6:**
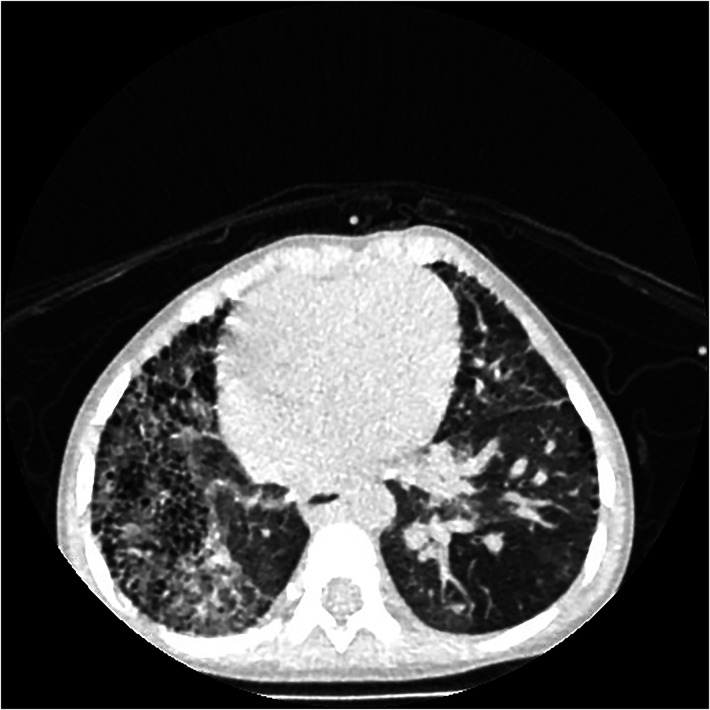
Right proximal interruption of the pulmonary artery with hemoptysis (twice) in a 22-month-old male patient. A transverse view of a plain computed tomography scan shows speckled and patchy shadows in the right lung field, and reticulated shadows, thickened interlobular septa, and small subpleural cystic lucency shadows were observed in both lungs.

If PIPA is complicated by collateral vessel rupture and hemorrhage, the lung field lucency on the affected side will be decreased, and the lung field will be scattered with speckled or patchy, fuzzy shadows (see Figure 6). Furthermore, thin, reticular shadows will be seen in the periphery of the affected lung, which is caused by the collateral branches between the systemic circulation and the pulmonary circulation vessels, as well as post-inflammatory fibrosis (3). Computed tomography will make it easy to observe shadows in the affected lungs of patients with a pulmonary artery interruption in collateral vessels (see Figure 6).

#### Subpleural cystic lucency shadow, subpleural parenchyma, pleural serrated thickening, and the mosaic sign

**Figure 7:**
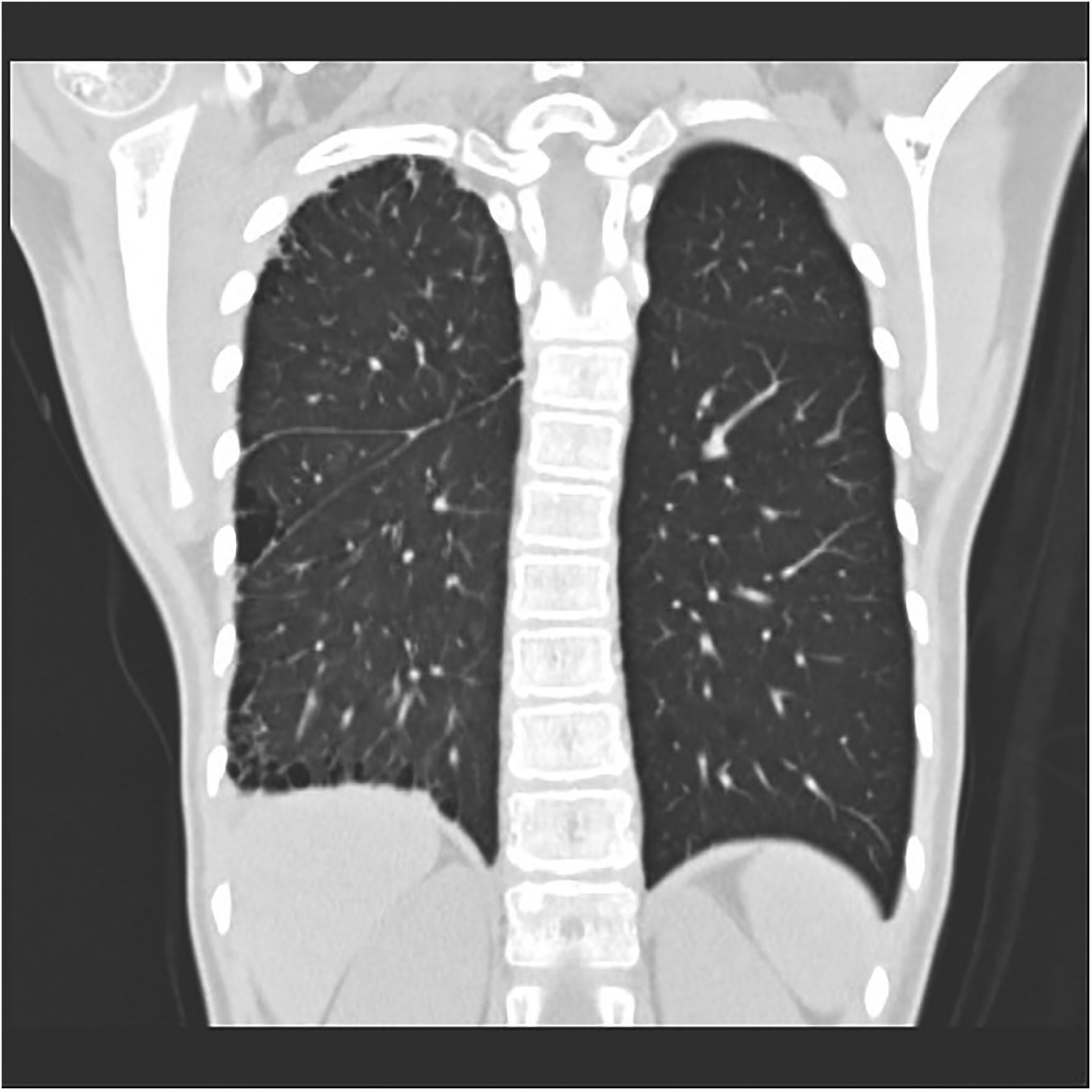
Right proximal interruption of the pulmonary artery in a 4-year-old female patient. A coronal view of a plain computed tomography scan shows a serrated pleural shadow in the right lung with a subpleural cystic lucency shadow.

Subpleural cystic changes that are secondary to chronic infection or ischemia (see Figure 7), bronchiectasis, potential rib notches, and pleural thickening associated with collateral vasculogenesis may be present (15) in patients with PIPA but are more common in older children or adults.

**Figure 8:**
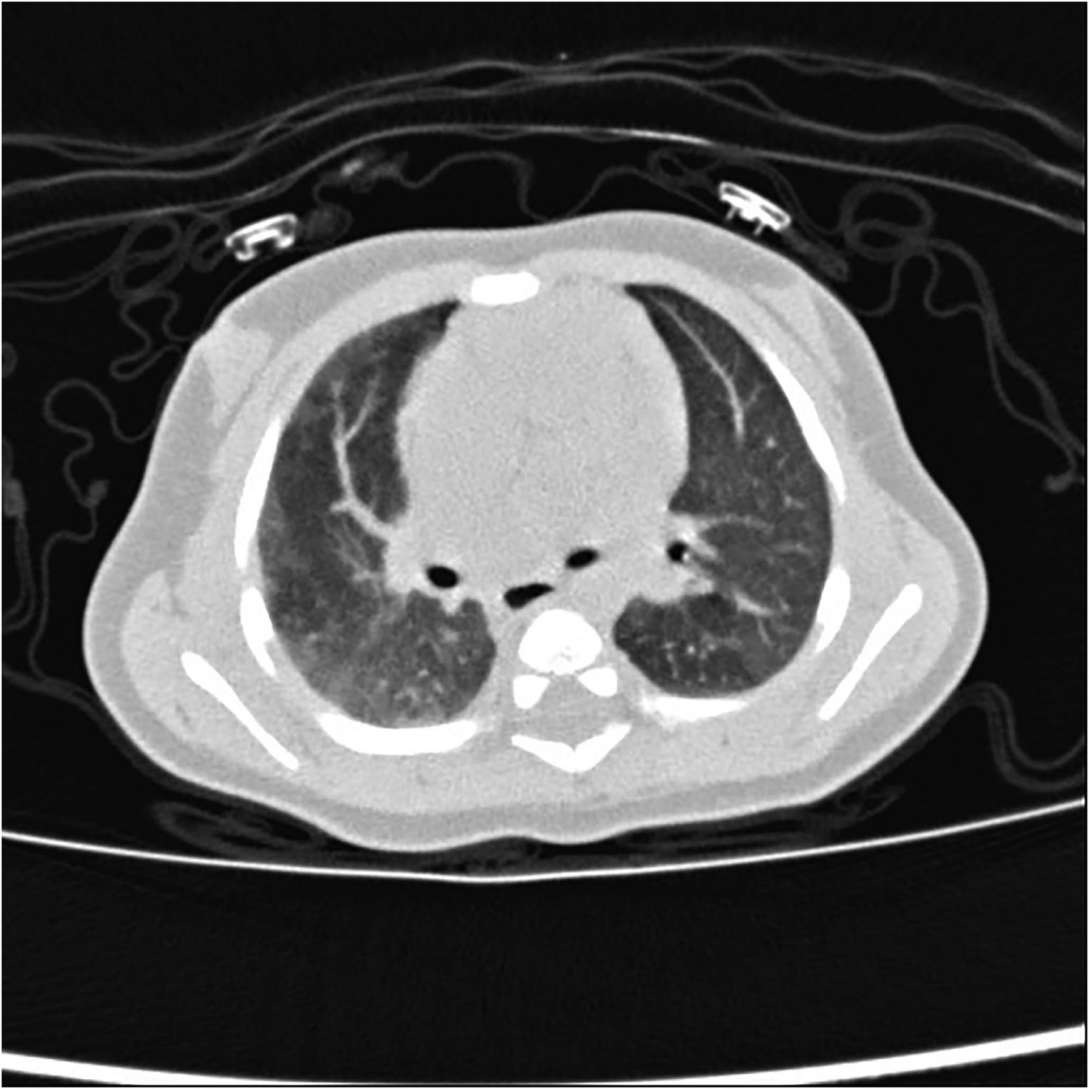
Right proximal interruption of the pulmonary artery in a 1-month-old male patient. A transverse view of a plain computed tomography scan shows strip-like shadows of subpleural pulmonary parenchyma along the periphery of the right lung at ground-glass opacity and a mild mosaic sign in the left lung.

In patients with PIPA, the bronchial artery and the pleural systemic collateral arteries can supply blood to the affected pulmonary artery. If the bronchial artery collateral supply is insufficient, the transpleural systemic circulation collateral arteries may expand and anastomose directly with the pulmonary artery branches, resulting in serrated pleural thickening (see Figure 7) and subpleural parenchyma (see Figure 8) (24, 42, 43).

The asymmetry of blood flow in both lungs is considered to be the cause of the mosaic sign, which indicates the likelihood of pulmonary hypertension (see Figure 8) (42, 44). The causes of the mosaic sign in the unaffected lung include air retention, compensatory hyperinflation, and increased pulmonary blood flow. Repeated infections are also an important cause of the subpleural parenchymal shadow and the mosaic sign. The causes of the mosaic sign in the affected lung may include reduced pulmonary blood flow and infection.

#### The significance of proximal interruption of the pulmonary artery and the relative position of the aortic arch

Most cases of PIPA are located contralateral to the aortic arch, but a few are located ipsilateral to the aortic arch (see Figure 2C). In the present study, 22 cases of PIPA were found contralateral to the aortic arch, accounting for 88% (22/25) of cases, and three PIPA cases were found ipsilateral to the aortic arch, accounting for 12% (3/25) of cases.

The present study group included 8 cases of co-occurring cardiovascular abnormalities, including 1 case of right-sided PIPA with a right-sided aortic arch, 1 case of left-sided PIPA with a left-sided aortic arch, 1 case of left-sided PIPA with a right-sided aortic arch, and 5 cases of right-sided PIPA with a left-sided aortic arch. The incidence of cardiovascular abnormalities in patients with PIPA and an ipsilateral aortic arch was 66.7% (2/3), and in patients with PIPA and a contralateral aortic arch, this was 27.3% (6/22). These findings suggested that the incidence of other cardiovascular abnormalities in patients with PIPA that were located ipsilateral to the aortic arch was much higher than in patients with PIPA located contralateral to the aortic arch (66.7% vs. 27.3%). Among 8 patients with cardiovascular abnormalities (CAVC/DORV/SV/PS), the most severe complication related to these occurred in patients with right-sided PIPA and a right-sided aortic arch; this indicated that right-sided PIPA with a right-sided aortic arch was highly likely to be complicated by other cardiovascular abnormalities and that these abnormalities were also the most serious type to co-occur with PIPA.

## Conclusion

It is more appropriate to refer to the absence of the pulmonary artery as PIPA. The clinical manifestations of patients with PIPA are not specific or asymptomatic, and the possible symptoms include chronic cough, recurrent pulmonary infections, SBE, and hemoptysis.

Although right-sided PIPA is more common, left-sided PIPA is not uncommon. The number of cases with PIPA located contralateral to the aortic arch is higher than for PIPA located ipsilateral to the aortic arch; in the latter, patients are significantly more likely to be complicated by other cardiovascular abnormalities than those with PIPA located contralateral to the aortic arch, while patients with right-sided PIPA and a right-sided aortic arch tend to be complicated by the most complex and severe cardiovascular abnormalities.

Computed tomography angiography can be used to determine PIPA and display the number, origin, distribution, interruption location, and distance from the hilus pulmonis. It can also show abnormalities in the trachea–bronchial tree, lung parenchyma, and pleura, and identify any co-occurring cardiovascular abnormalities, which is extremely helpful for the design of a suitable therapeutic regimen.

The prognosis of patients with PIPA is associated with the severity of the accompanying cardiovascular abnormalities and pulmonary hypertension, which are the most decisive factors. Early diagnosis can prevent severe complications, such as massive hemoptysis and severe pulmonary hypertension, thereby providing strong support for surgical treatment or medication.

## Data Availability

The original contributions presented in the study are included in the article/Supplementary Material, further inquiries can be directed to the corresponding author/s.
